# Transdiagnostic Biomarkers of Psychopathology: A Conceptual Review

**DOI:** 10.1111/eip.70267

**Published:** 2026-10-01

**Authors:** Katelyn Hummel, Luigi Boccuto, Sara M. Sarasua, Kendra Allison, Garrie B. Thompson

**Affiliations:** ^1^ Healthcare Genetics and Genomics Program, School of Nursing, College of Behavioral, Social and Health Sciences Clemson University Clemson South Carolina USA; ^2^ School of Nursing, College of Behavioral, Social and Health Sciences Clemson University Clemson South Carolina USA; ^3^ Independent Clinical Neuropsychologist Hendersonville North Carolina USA

**Keywords:** conceptual review, early intervention, psychiatric biomarkers, psychopathology, transdiagnostic

## Abstract

**Introduction:**

Psychiatric biomarker research has traditionally been organized around diagnostic categories, but clinical heterogeneity, comorbidity and shared biological mechanisms across psychiatric conditions have increased interest in transdiagnostic approaches. These approaches may be especially relevant for early recognition, stratification and intervention planning. Despite this growing interest, the concept of the transdiagnostic biomarker remains unclear. This conceptual review aimed to clarify how transdiagnostic biomarkers of psychopathology are represented in psychiatric research.

**Methods:**

Systematically identified empirical literature and concept explication were used to examine biomarker modalities, indexed psychopathology‐related constructs, transdiagnostic framing and conceptual boundary issues across studies.

**Results:**

Transdiagnostic biomarker research was found to encompass several approaches, including diagnosis‐spanning comparisons, symptom‐based models, subgroup‐based approaches, cross‐domain extensions beyond traditional psychiatric categories and research aligned with alternative frameworks such as the Research Domain Criteria or the Hierarchical Taxonomy of Psychopathology. A conceptual framework is proposed comprising three linked elements: the measurable indicator, the psychopathology‐related construct and the transdiagnostic scope of that construct. Within this framework, a transdiagnostic biomarker is defined as a measurable biological or biobehavioural indicator of a psychopathology‐related construct that spans multiple diagnostic categories or extends beyond them.

**Conclusion:**

Greater conceptual clarity of transdiagnostic biomarkers may strengthen psychiatric biomarker discovery by improving construct alignment and supporting their use in biomarker‐informed stratification, early intervention, treatment selection, prognosis and precision psychiatry.

## 1 Introduction

Diagnosis has long served as the central organizing principle of clinical medicine and scientific classification, and psychiatry has adopted this tradition through formal nosologies that structure research, treatment and clinical decision‐making (van den Tweel and Taylor 2010). Modern psychiatric diagnosis emerged from classification systems influenced by broader natural science traditions and became institutionalized through the Diagnostic and Statistical Manual of Mental Disorders (DSM) and the International Classification of Diseases (ICD), which continue to function as the dominant frameworks for defining mental disorders in research and practice (Kendler 2009, 2018; Lawrie et al. 2016). These systems have offered a common language for describing mental illness and have shaped how clinicians decide when to treat, how researchers define samples and how evidence is generated and interpreted.

At the same time, there is increasing concern that diagnosis‐based psychiatry may have reached the limits of its research and clinical utility. Although successive DSM and ICD revisions improved reliability and psychometric precision, they remain fundamentally descriptive systems based on self‐reported experience and observed behaviour rather than on validated biological mechanisms (Insel and Cuthbert 2015; Kozak and Cuthbert 2016). This creates a central problem for biomarker research. If psychiatric diagnoses are not biologically grounded, it becomes difficult to expect clear biological tests to map neatly onto them. In this sense, psychiatry has been described as being in a ‘catch‐22’, where current classification systems are poorly suited to biological differentiation, yet biologically grounded alternatives have not matured into viable clinical replacements (Kapur et al. 2012).

Several related problems intensify this difficulty. First, psychiatric symptoms often appear to vary along continuous dimensions rather than existing as all‐or‐none disease entities (Allardyce et al. 2007; Andrews et al. 2007; Haslam et al. 2012). Threshold‐based diagnosis imposes binary judgements on phenomena that are often graded, unstable and context‐sensitive (Markon et al. 2011). Second, psychiatric disorders show extensive comorbidity, such that overlapping diagnoses are often more common than isolated, single‐disorder presentations (Kessler et al. 2005, 2008, 2015; Shi et al. 2023). This overlap raises the possibility that at least some diagnostic distinctions are artefacts of the classification system rather than evidence of truly separate syndromes (Maj 2005; van Loo and Romeijn 2015). Third, there is marked heterogeneity within disorders. Individuals who meet criteria for the same diagnosis may share relatively few symptoms, making it difficult to identify specific biological correlates of any one diagnostic category (Fried and Nesse 2015; Galatzer‐Levy and Bryant 2013; Olbert et al. 2014). Finally, diagnostic systems may capture only a subset of clinically relevant symptom variation, further complicating efforts to align biology with diagnosis (Fried 2017; Santor and Gregus 2006).

Historically, psychiatric research has tended to examine one disorder at a time, often contrasting a single diagnostic group with healthy controls, even though the lived reality of psychopathology rarely conforms to such clean boundaries (Barch 2020). Risk factors are likewise often studied within single disorders, despite evidence that many may operate across multiple, highly comorbid conditions (Aldao et al. 2010; Buckholtz and Meyer‐Lindenberg 2012; Shi et al. 2023). In treatment research, diagnosis‐specific protocols dominate the evidence base, yet many commonly used interventions appear to work across broad clinical populations, and clinicians frequently adapt treatment pragmatically to the individual rather than the diagnosis alone (Dalgleish et al. 2020). Together, these concerns have contributed to growing support for approaches that move beyond disorder‐by‐disorder models.

Transdiagnostic approaches have gained prominence as alternatives to traditional diagnostic thinking. Rather than assuming that each disorder has its own distinct causes, mechanisms and treatments, transdiagnostic approaches emphasize dimensions, processes, vulnerabilities and mechanisms that cut across conventional diagnostic categories (Buckholtz and Meyer‐Lindenberg 2012; Dalgleish et al. 2020). This shift has been reinforced by a number of influential frameworks. The Research Domain Criteria (RDoC) initiative was introduced to encourage research on dimensions of observable behaviour and neurobiological function that cross diagnostic boundaries (Cuthbert 2014; Cuthbert and Insel 2013; Insel 2014). Similarly, the Hierarchical Taxonomy of Psychopathology (HiTOP) was developed to address heterogeneity, comorbidity and arbitrary diagnostic boundaries through a data‐driven hierarchy of psychopathology spectra and subfactors (Kotov et al. 2017, 2018; Waszczuk et al. 2020). Related efforts, such as the clinical staging model, also seek to represent psychopathology in ways that better capture progression, overlap and shared vulnerability across conditions (Scott et al. 2013). These developments mark a broader transition from diagnosis‐centred models towards frameworks organized around shared dimensions, vulnerabilities and mechanisms of psychopathology, providing the conceptual foundation on which transdiagnostic research would later emerge.

This transition is particularly relevant to early intervention in psychiatry, where clinical presentations are often nonspecific, developmentally evolving and overlapping across diagnostic categories. Individuals may initially present with subthreshold symptoms, mixed affective and psychotic features or functional decline before a stable diagnosis is established. In this context, transdiagnostic frameworks may be useful not because they replace diagnosis, but because they provide a way to study shared risk, onset, progression, prognosis and intervention response during phases when categorical boundaries are least stable. This makes transdiagnostic biomarker research particularly relevant to efforts aimed at earlier recognition, stratification and intervention across mental and substance use disorders.

### 1.1 Historical Development of the Term ‘Transdiagnostic’

#### 1.1.1 Transdiagnostic Treatment Approach

The term *transdiagnostic* first gained traction within the context of psychological treatment, for example, in cognitive‐behavioural therapy (CBT). Early transdiagnostic interventions were developed first for eating disorders and later included anxiety and depressive disorders (Clark 2009; Fairburn 2008; Norton and Philipp 2008). These early treatment approaches were motivated by the observation that many common mental disorders appeared to share overlapping etiological, maintenance, interpersonal, cognitive, affective and behavioural features, suggesting that disorder‐specific treatment models might be overlooking broader mechanisms that operate across diagnoses (Caspi et al. 2014; Harvey et al. 2004). Within the CBT literature, the transdiagnostic approach was framed as an alternative to the assumption that each disorder requires its own separate explanatory model and intervention. Proponents argued that multiple psychological disorders may be maintained by a common set of cognitive and behavioural processes, and that these shared processes may be more useful therapeutic targets than diagnosis‐specific symptoms alone (Harvey et al. 2004). In this view, diagnostic assessment is not always necessary for effective intervention if the relevant cross‐cutting mechanisms can be identified and addressed directly (Mansell et al. 2009). The transdiagnostic treatment approach therefore proposed a shift in emphasis from treating discrete diagnostic entities to targeting broadly operating mechanisms of illness.

Importantly, even in these early treatment discussions, the transdiagnostic idea was never limited to psychology alone. Authors within this practice explicitly suggested that transdiagnostic processes might exist at multiple levels of explanation, including biology, genetics, emotion, personality, family systems and social context (Kendler and Prescott 2006; Mansell et al. 2009). This broader framing foreshadowed the later migration of transdiagnostic language from psychotherapy into psychiatric research.

#### 1.1.2 Transdiagnostic Research Approach

As transdiagnostic thinking expanded beyond treatment approaches, it began to function as a research framework. In this newer usage, the term increasingly referred to studies that attempted to examine mechanisms, symptoms, traits or biological features across, rather than within, diagnoses. This expansion was conceptually attractive because it aligned with growing dissatisfaction with diagnosis‐centred research, but it also introduced substantial inconsistency in how the term was applied.

A systematic review of transdiagnostic psychiatry by Fusar‐Poli et al. (2019) illustrates both the expansion and the instability of the concept. Across 111 studies, most self‐identified transdiagnostic papers focused on interventions, followed by cognition, psychological processes and neuroscientific topics (Fusar‐Poli et al. 2019). However, the authors found marked methodological inconsistency in how studies defined diagnostic standards, outcomes and the term *transdiagnostic* itself. Most remained close to traditional DSM categories, using what the authors described as an ‘across‐diagnoses’ model in which multiple diagnostic groups were compared to identify shared or cross‐cutting features. Only a minority of studies moved ‘beyond diagnoses’ by proposing or testing novel constructs, such as clinical or biological subtypes, rather than simply comparing existing categories. No universal transdiagnostic construct was identified across disorders or spectra. Instead, the literature appeared conceptually loose, with the label applied to studies that differed substantially in design and scope.

### 1.2 Biomarkers in Psychiatry

A biomarker has been defined as a characteristic that is objectively measured and evaluated as an indicator of normal biological processes, pathogenic processes or biological responses to an intervention (Biomarkers Definitions Working Group 2001). The search for biomarkers in psychiatry has been driven by the hope that these objective biological indicators might improve diagnosis, prognosis, treatment selection and monitoring (García‐Gutiérrez et al. 2020). This broader role is especially relevant to early intervention research, where biomarkers may be useful as indicators of risk or early‐course monitoring. In principle, biomarkers may include genes, proteins, other biomolecules, physiological responses, imaging features or morphological characteristics.

Despite their promise, clinically useful biomarkers in psychiatry remain limited (Venkatasubramanian and Keshavan 2016). One reason for this is that biomarker research has largely been organized around traditional diagnostic categories, even though those categories are marked by heterogeneity within disorders and overlap across them. At the genetic level, psychiatric disorders show substantial polygenicity and pleiotropy, with many variants and polygenic risk scores indexing broad cross‐disorder vulnerability rather than diagnosis‐specific liability (Cross‐Disorder Group of the Psychiatric Genomics Consortium 2013a, 2013b; Hoy et al. 2023; Waszczuk et al. 2023). As a result, biological differences observed at the group level have rarely translated into clinically useful markers (Venkatasubramanian and Keshavan 2016). This may reflect a mismatch between expectations for diagnostic specificity and the broader biological signals observed across disorders, which may be better suited to identifying general risk than supporting diagnostic classification. These limitations have made transdiagnostic frameworks especially appealing for biomarker research. If central biological processes cut across conventional diagnoses, then biomarkers linked to shared symptom dimensions or general liability may be more informative than those tied to disorder‐specific categories (McQuaid 2021; Michelini et al. 2021).

### 1.3 The Emergence of the Transdiagnostic Biomarker Concept

As frameworks such as RDoC and HiTOP gained influence, they provided alternative targets for biomarker research by emphasizing shared mechanisms, phenotypic continua and cross‐cutting domains of psychopathology (Cuthbert 2014; Kotov et al. 2018). In this context, transdiagnostic biomarkers have been proposed as indicators of shared symptom dimensions, broader psychopathology spectra, data‐driven subtypes, burden, vulnerability or treatment response. However, this expansion has also introduced conceptual ambiguity. Empirical research depends on adequately delineated concepts, as poorly defined or misconceived concepts can lead to inappropriate research questions, methods and interpretations that empirical analysis alone cannot resolve (Marková and Berrios 2016). This is particularly relevant in psychiatry, where the boundaries of its objects of study are complex and historically constructed. Accordingly, the term *transdiagnostic* is now applied across a range of biomarker contexts without a consistently articulated understanding of what qualifies a biomarker as transdiagnostic. The present conceptual review examines patterns of use in the existing literature to clarify the concept and develop a framework to guide future psychiatric biomarker research.

## 2 Methods

This study was designed as a conceptual review grounded in McLeod and Pan's framework for concept explication, which offers a systematic approach for clarifying the relationship between a concept's meaning and its empirical or operational manifestations (McLeod and Pan 2005). Concept explication is central to conceptual article writing, providing the foundation for developing and organizing a clear conceptual argument (Reese 2023). This approach was used to examine how the transdiagnostic biomarker is defined, operationalized and bounded in the literature, with the goal of developing a conceptual framework for future psychiatric biomarker research.

To identify relevant literature, a systematic search was conducted in Web of Science and PubMed through March 5, 2026, with no restrictions on language or publication date. The Web of Science search included all available databases. The terms ‘transdiagnostic’ (Title) AND ‘biomarker*’ (Topic) were used, with results filtered to the Psychiatry category using the Web of Science categories function. PubMed was searched using the terms ‘transdiagnostic’ [Title] AND ‘Biomarkers’ [Mesh]. Together, these searches identified 155 records. After the removal of 38 duplicates, 117 titles and abstracts were screened.

Studies were eligible for inclusion if they were original individual articles and had a clear and primary focus on transdiagnostic biomarkers. Exclusion criteria were reviews, meta‐analyses, study protocols, abstracts, commentaries, news articles and any other nonoriginal research. Following title and abstract screening, 62 records were excluded, including editorials (*n* = 1), news articles (*n* = 1), conference abstracts (*n* = 8), books (*n* = 3), commentaries (*n* = 3), study protocols (*n* = 12), a foreign language publication (*n* = 1) and review articles (*n* = 33). Full texts of 55 articles were reviewed and 49 met the inclusion criteria for the conceptual review. Of the six excluded articles, one was not transdiagnostic in study design (Innis et al. 2009), three were more appropriately classified as studies of psychological or behavioural processes rather than biomarkers (Brenning et al. 2022; Contreras‐Huerta et al. 2022; Hestbaek et al. 2025), one examined a behavioural and methodological measure relevant to neuroimaging rather than a biomarker (Hercules et al. 2025), and one was a measurement tool development paper rather than a biomarker study (Correll et al. 2024). These articles were retained for conceptual discussion because they illustrate important boundary issues in the literature. The full study selection process is summarized in Figure 1.

**FIGURE 1:**
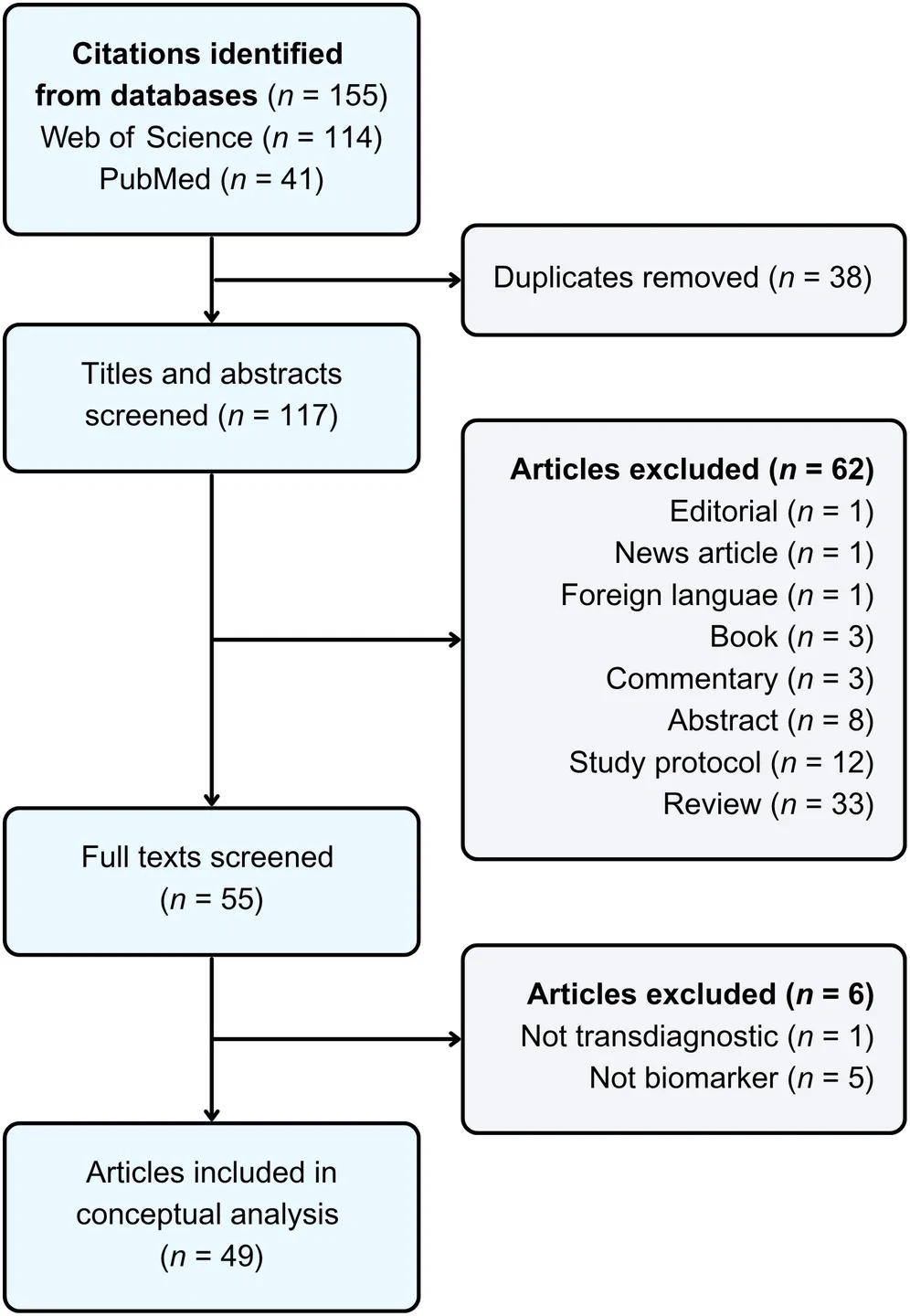
Flow diagram illustrating the study selection process. A total of 155 records were identified through database searches. After removing duplicates and screening titles, abstracts and full texts, 49 articles were included in the conceptual review.

For each included study, information was extracted on the biomarker type, the construct indexed by the biomarker, the type of transdiagnostic approach and how *transdiagnostic* was operationalized (see Table S1). Analysis proceeded in four steps: first, extracted features were grouped descriptively to characterize recurring patterns in biomarker type, indexed construct and transdiagnostic approach; second, operational uses of *transdiagnostic* were compared across studies; third, boundary cases and adjacent concepts were examined to clarify the limits of the concept and fourth, these patterns were synthesized into a conceptual framework for future psychiatric biomarker research.

## 3 Results

The final analytic sample comprised 49 studies published between 2016 and 2026, with most appearing from 2020 onward. As a whole, this literature showed substantial heterogeneity in the empirical and operational manifestations of the concept, including the type of biomarker examined, the constructs these biomarkers were intended to index, and how transdiagnostic relevance was established (Figure 2).

**FIGURE 2:**
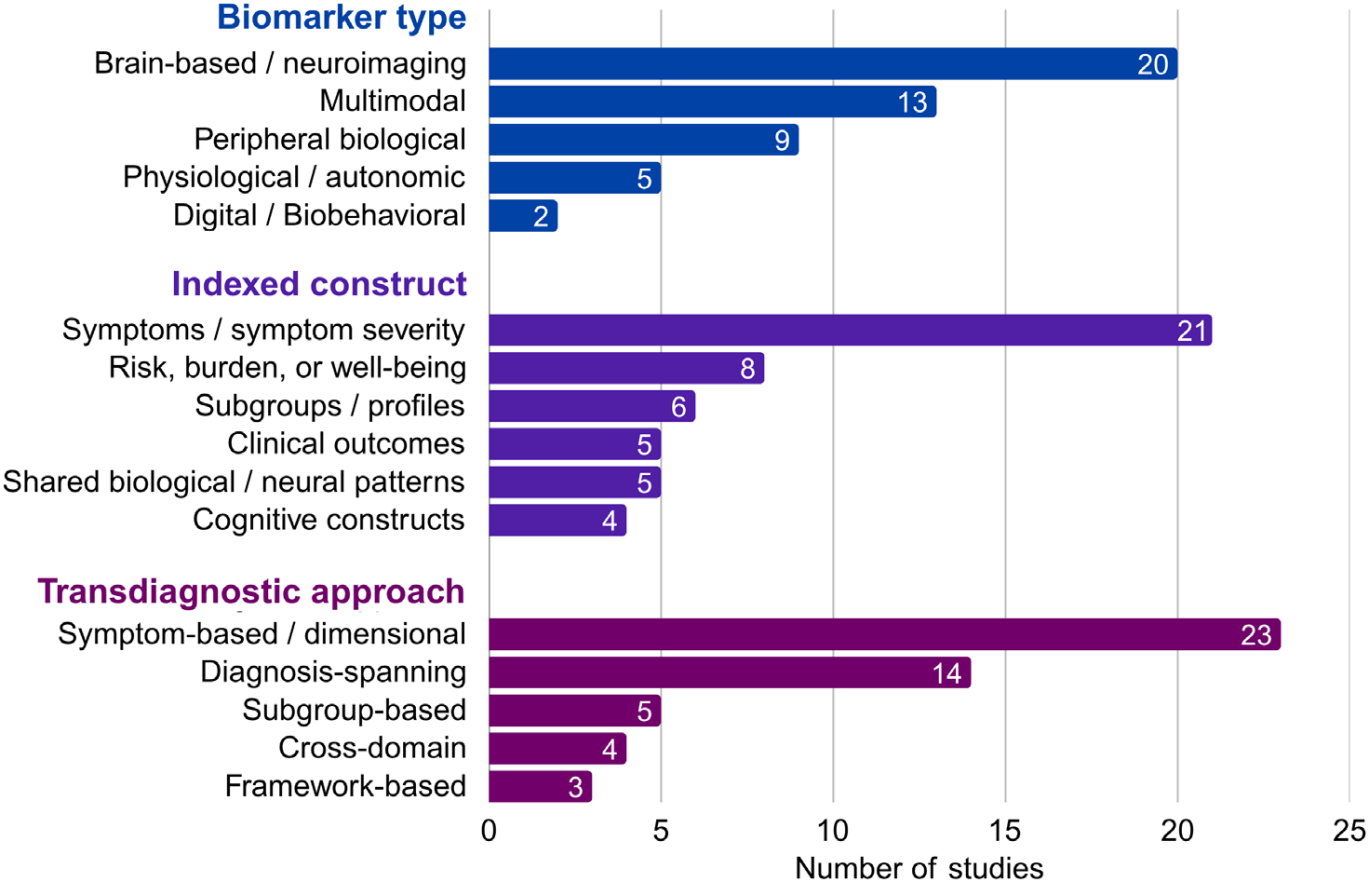
Descriptive groupings by biomarker type, indexed construct and transdiagnostic approach. Studies were grouped according to biomarker modality, the construct indexed by the biomarker and how *transdiagnostic* was operationalized.

### 3.1 Biomarker Types

A prominent pattern was the dominance of brain‐based studies. Neuroimaging papers formed a large proportion of the sample, including work on structural MRI, resting‐state functional connectivity, dynamic connectivity, connectome‐based prediction, graph theory metrics, brain iron distribution and electrophysiological markers integrated with neuroimaging approaches (Bruin et al. 2024; Chen et al. 2026; Coray et al. 2025; Fang et al. 2024; Feurer et al. 2022; Jia et al. 2022; Kim et al. 2023; Lalousis et al. 2022; Li et al. 2021, 2023; Meijs et al. 2025; Pang et al. 2018; Parkes et al. 2021; Qi et al. 2021; Randau et al. 2024; Rößler et al. 2025; Sun et al. 2025, 2026; Thomas et al. 2020; Tong et al. 2022). Another large subset used multimodal designs, combining imaging with endocrine, inflammatory, cognitive, microbiome, genomic or psychological indicators (Cornblath et al. 2020; Den Ouden et al. 2022; Hüper et al. 2024; Lee et al. 2023; López‐Villatoro et al. 2024; McIntosh et al. 2022; Mellem et al. 2020; Sánchez‐Ortí et al. 2024; Wang et al. 2025; Wei et al. 2023; Zhang et al. 2022). A few studies centred on unconventional biomarkers, including actigraphy‐derived sleep and activity patterns and AI‐derived linguistic features, while another combined behavioural measures of VR‐elicited emotional reactivity with galvanic skin response as a psychophysiological indicator (Hamitouche et al. 2025; Hercules et al. 2025; Kjærstad et al. 2025; Themistocleous and Stark 2026). Together, these studies suggest transdiagnostic biomarker research is often presented as a broader effort to integrate multiple levels of analysis.

Another major subgroup involved peripheral biological markers, such as inflammatory, endocrine, metabolic, genomic and molecular indicators. These included BDNF and cell adhesion molecules, CRP, oxytocin, proteomic signatures, epigenetic clocks, cytokines, telomere‐related measures, PACAP and inflammatory/oxidative components (Byrne et al. 2022; Levchuk et al. 2020; Orsolini et al. 2025; Rietberg et al. 2026; Ross et al. 2020; Seitz‐Holland et al. 2025; Shemesh et al. 2024; Soloey‐Nilsen et al. 2024; Xu et al. 2020; Yusupov et al. 2023). Within this subgroup, inflammation appeared especially prominent, recurring across mood disorders, psychosis‐spectrum conditions, severe mental illness, self‐injurious behaviour, executive dysfunction and psychiatric burden (Byrne et al. 2022; López‐Villatoro et al. 2024; McIntosh et al. 2022; Orsolini et al. 2025; Rietberg et al. 2026; Wang et al. 2025; Wei et al. 2023). A smaller subset used primarily physiological and electrophysiological markers, including heart rate variability, niacin skin flushing response and other autonomic indicators (Chalmers et al. 2016; Feng et al. 2026; Qing et al. 2022; Rüesch et al. 2023; Wen et al. 2024).

### 3.2 Indexed Constructs

The indices of these biomarkers varied considerably. Many studies focused on cross‐cutting symptom dimensions or symptom severity, including worry, anhedonia, suicidality, anger‐hostility, executive dysfunction, general distress, psychosis‐related symptoms and depression/anxiety dimensions (Chalmers et al. 2016; Levchuk et al. 2020; Li et al. 2023; McIntosh et al. 2022; Mellem et al. 2020; Orsolini et al. 2025; Parkes et al. 2021; Rüesch et al. 2023; Soloey‐Nilsen et al. 2024; Sun et al. 2026; Wang et al. 2025; Wei et al. 2023; Wen et al. 2024; Xu et al. 2020). In these studies, biomarkers were presented as indexing constructs that cut across diagnoses rather than marking a single categorical disorder. This symptom‐centred usage was among the clearest expressions of transdiagnostic biomarker research in the sample. Several studies also indexed broader cross‐diagnostic subgroups or profiles through clustering, machine learning or biologically defined stratification. In these studies, biomarkers were used to define, validate or predict groupings that did not map neatly onto DSM categories or individual symptoms (Byrne et al. 2022; Cornblath et al. 2020; Den Ouden et al. 2022; Kim et al. 2023; Lalousis et al. 2022; Zhang et al. 2022). Other studies indexed treatment response, prognosis, risk or stratification rather than baseline symptom dimensions. Examples included biomarkers associated with CBT or antidepressant response, psychotherapy outcomes across disorders, remission or poor outcome, familial risk, neuroanatomical vulnerability, psychiatric disease burden and targeted intervention (Coray et al. 2025; Fang et al. 2024; Feurer et al. 2022; Jia et al. 2022; Lalousis et al. 2022; Meijs et al. 2025; Qi et al. 2021; Rietberg et al. 2026; Rößler et al. 2025; Thomas et al. 2020; Yusupov et al. 2023).

### 3.3 Transdiagnostic Framing and Operationalization

The literature also differed in how *transdiagnostic* was operationalized. The most common approach was diagnosis‐spanning research, in which biomarkers were examined across multiple categorical diagnoses to identify shared or overlapping features (Chen et al. 2026; Coray et al. 2025; Feurer et al. 2022; Jia et al. 2022; Kjærstad et al. 2025; Li et al. 2021; Qing et al. 2022; Rietberg et al. 2026; Rößler et al. 2025; Seitz‐Holland et al. 2025; Shemesh et al. 2024). Within this approach, some studies had a narrower transdiagnostic scope, examining biomarker patterns across only two diagnostic groups (Feng et al. 2026; Pang et al. 2018; Sun et al. 2025). A second major approach was symptom‐based or dimensional, in which biomarkers were examined in relation to cross‐cutting symptoms, severity measures or dimensional psychopathology, decentring diagnosis entirely. This approach was evident in studies of worry, anhedonia, executive dysfunction, general distress, anger‐hostility, novelty seeking, suicidality, depressive/anxious symptom dimensions and overall psychiatric disease burden (Chalmers et al. 2016; Levchuk et al. 2020; Li et al. 2023; Mellem et al. 2020; Orsolini et al. 2025; Qi et al. 2021; Soloey‐Nilsen et al. 2024; Sun et al. 2026; Wen et al. 2024; Yusupov et al. 2023). A smaller subset of studies used subgroup‐based approaches, deriving clusters, latent profiles or biologically defined subgroups that cross traditional diagnostic boundaries (Byrne et al. 2022; Cornblath et al. 2020; Den Ouden et al. 2022; Kim et al. 2023; Lalousis et al. 2022; Zhang et al. 2022). Only a few studies explicitly anchored their approach in preexisting transdiagnostic frameworks, including p‐factor, HiTOP and RDoC. Parkes et al. (2021) used a p‐factor‐informed dimensional structure, Randau et al. (2024) mapped ERP findings onto the HiTOP hierarchy, and Meijs et al. (2025) framed frontal beta activity within RDoC arousal/regulatory systems. Although relatively few, these studies are important because they anchor transdiagnostic biomarker research in groundbreaking conceptual models of psychopathology.

Several studies combined transdiagnostic and differential‐diagnostic aims, using the same biomarker to examine both shared and disorder‐specific features (Chen et al. 2026; Feng et al. 2026; Kjærstad et al. 2025; Pang et al. 2018; Sun et al. 2025). Some also drew on related constructs such as endophenotypes (Jia et al. 2022; López‐Villatoro et al. 2024). Others extended transdiagnostic biomarker research beyond psychiatry by including medical, neurological or neurodegenerative conditions (Cornblath et al. 2020; Hüper et al. 2024; Rößler et al. 2025; Sánchez‐Ortí et al. 2024; Themistocleous and Stark 2026).

## 4 Discussion

### 4.1 A Conceptual Framework for Transdiagnostic Biomarkers

The reviewed literature suggests that the term *transdiagnostic biomarker* is not used in a single uniform way. Across the included studies, it referred variously to biomarkers examined across two or more diagnoses, markers linked to shared symptom dimensions, biomarker‐informed phenotypes or subtypes, and indicators of burden, prognosis, vulnerability or treatment response. Consistent with prior observations about transdiagnostic research more broadly, these uses reflect both of its root meanings: across and beyond diagnoses (Definition of TRANS 2026; Fusar‐Poli et al. 2019).

Based on these patterns, we propose a conceptual framework organized around three linked elements: the measurable indicator, the construct indexed by that indicator and the transdiagnostic scope of that construct (Figure 3). The first concerns what is being measured as a biomarker, such as a biological or biobehavioural characteristic. The second concerns what the biomarker is intended to represent, which may include a symptom dimension, mechanism, vulnerability, liability, phenotype, profile or clinically relevant outcome. The third concerns how that construct relates to conventional diagnostic categories. Transdiagnostic theory has previously been described as seeking to identify factors or constructs that contribute to the onset and/or maintenance of multiple disorders (Fernandez et al. 2016). The present review suggests that these factors may be examined across diagnostic categories or conceptualized independently of them. Accordingly, the transdiagnostic character of a biomarker depends not simply on its inclusion in a study involving more than one diagnosis, but on whether it indexes a feature of psychopathology that is itself transdiagnostic. Within this framework, a transdiagnostic biomarker can be understood as a measurable biological or biobehavioural indicator of a psychopathology‐related construct that spans multiple diagnostic categories or extends beyond them. The construct may be relatively narrow, such as worry, anhedonia, suicidality or executive dysfunction, or broader, such as general psychopathology liability, a biomarker‐informed subgroup or a cross‐diagnostic treatment‐response profile.

**FIGURE 3:**
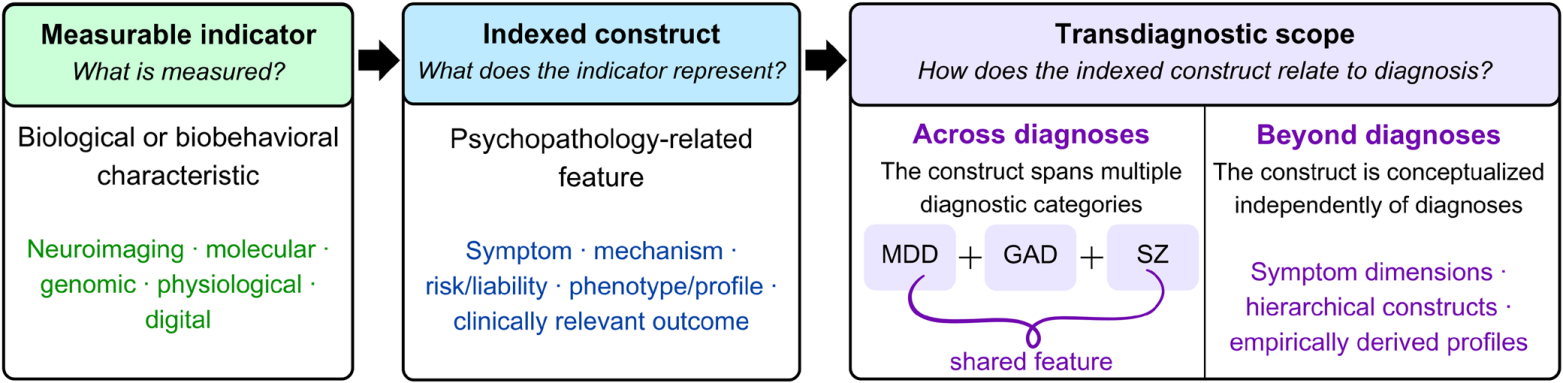
Conceptual framework for transdiagnostic biomarkers. The framework is organized around three linked elements: the measurable indicator, the psychopathology‐related construct indexed by that indicator, and the transdiagnostic scope of that construct. Transdiagnostic scope may be expressed across diagnoses when a shared feature spans multiple diagnostic categories or beyond diagnoses when the construct is conceptualized independently of conventional diagnostic categories. Examples shown within each box are illustrative rather than exhaustive and are intended to demonstrate the range of biomarker modalities, indexed features and transdiagnostic formulations represented in the literature. GAD, generalized anxiety disorder; MDD, major depressive disorder; SZ, schizophrenia.

This distinction also helps clarify the relationship between different transdiagnostic research designs. In across‐diagnoses approaches, diagnostic categories remain part of the study design, but the aim is to identify biological features or biomarker–construct relationships that are shared across those categories. In beyond‐diagnoses approaches, diagnosis is further decentred in favour of symptom dimensions, hierarchical constructs or empirically derived profiles that are not organized around conventional diagnostic boundaries. This may include presentations in which a clear categorical disorder has not yet emerged, but symptoms, vulnerabilities or other psychopathology‐related features are already evident. This distinction parallels the ‘across‐diagnoses’ and ‘beyond‐diagnoses’ approaches described by Fusar‐Poli et al. (2019) and the ‘soft’ and ‘hard’ transdiagnostic approaches discussed by Dalgleish et al. (2020). Both approaches may therefore support transdiagnostic biomarker research, provided that the biomarker is interpreted in relation to a construct that meaningfully crosses or decouples from categorical diagnosis.

The conceptual boundaries of the concept are further illustrated by studies identified in the search that used transdiagnostic or biomarker language but did not clearly satisfy both aspects of the term. Some studies examined biological markers without meaningfully extending across or beyond diagnostic categories, whereas others adopted a transdiagnostic approach but did not include a measure that qualified as a biomarker. Innis et al. (2009) examined plasma homocysteine and methionine as biological markers but did so within subtypes of anorexia nervosa. *Transdiagnostic* referred to the theoretical model being tested rather than to a biomarker that indexed a construct spanning or extending beyond diagnostic categories. Brenning et al. (2022) examined emotion regulation and psychological need experiences as transdiagnostic psychological processes, Contreras‐Huerta et al. (2022) examined affective characteristics associated with prosocial behaviour, and Hestbaek et al. (2025) examined transdiagnostic differences in emotional cognition using behavioural and performance‐based measures. These may function as transdiagnostic factors or outcomes but are not themselves biological or biobehavioural indicators. Similarly, Hercules et al. (2025) examined associations between transdiagnostic symptom dimensions and in‐scanner head motion. Although the study was transdiagnostic in its symptom‐based approach, head motion functioned primarily as a behavioural and methodological measure relevant to neuroimaging rather than as an indicator of an underlying biological or biobehavioural process. Finally, Correll et al. (2024) developed a clinical assessment tool for measuring transdiagnostic symptom severity. In this case, the instrument measures psychopathology directly rather than serving as a biomarker that indexes an underlying biological or biobehavioural characteristic.

These boundary cases clarify that a transdiagnostic psychological factor, behavioural or performance measure, or clinical assessment tool is not necessarily a transdiagnostic biomarker, just as a biological marker is not transdiagnostic when it reflects variation confined to a single diagnostic category. At the same time, behavioural measures should not be categorically excluded from biomarker research. Digital biomarker research illustrates how objectively captured behavioural characteristics may be evaluated as biomarkers when they function as indicators of biological or pathological processes (Coravos et al. 2019). Therefore, the distinction between a behavioural measure and a biobehavioural biomarker depends on how the measure functions and is interpreted. The behavioural boundary cases primarily used psychological, behavioural or performance‐based assessments that measured the phenotype itself rather than indexing underlying biological or pathological processes. Together, these boundary cases sharpen the definition of the transdiagnostic biomarker and clarify how the concept should be applied in future research.

The concept of the transdiagnostic biomarker overlaps with a few adjacent concepts. One important comparison is the endophenotype, a subset of biomarkers characterized by additional criteria including heritability, state dependence and association with illness (Beauchaine 2009; Gottesman and Gould 2003). Although endophenotypes were traditionally conceptualized in relation to specific psychiatric conditions (Flint and Munafò 2007; Waszczuk et al. 2023), more recent work has relaxed the expectation of disorder specificity to accommodate transdiagnostic vulnerabilities (Beauchaine and Constantino 2017; Kravariti et al. 2025). Therefore, some endophenotypes may be transdiagnostic biomarkers, but transdiagnostic biomarkers do not necessarily meet endophenotype criteria. A second adjacent concept is stratified psychiatry, which seeks to identify clinically meaningful subgroups using biological, cognitive, psychological or clinical measures (Kapur et al. 2012). Transdiagnostic biomarkers may contribute to such stratification, but neither stratified psychiatry itself nor all measures used for stratification are biomarkers. Together, these adjacent concepts help delineate the boundaries of the concept and distinguish it from related approaches in psychiatric research.

### 4.2 Implications and Recommendations for Future Research

The conceptual framework outlined above also has implications for the aims and applications of transdiagnostic biomarker research. The literature synthesized here supports the broader argument that transdiagnostic approaches may be especially useful for biomarker development in psychiatry. Biomarkers that map onto shared constructs across or beyond disorders may be better positioned to capture the heterogeneity and comorbidity that characterize real‐world psychopathology (McQuaid 2021). This is particularly important for precision psychiatry, which aims to develop more targeted and personalized interventions based on an individual's symptoms, biology and lived experience (Fernandes et al. 2017; Gandal et al. 2016; Serretti 2022). It may also be especially relevant early in the course of illness, when a categorical diagnosis has not yet emerged or remains fluid, subthreshold or mixed, while impairment and risk are clinically meaningful. The present study suggests that transdiagnostic biomarker research is already moving in this direction by linking measurable indicators to shared symptom dimensions, biomarker‐informed subtypes and cross‐diagnostic treatment‐response patterns. This reorients biomarker development towards targets that may be more stable, mechanistically informative and clinically actionable than diagnosis alone. The value of transdiagnostic biomarkers may lie less in confirming existing categories than in identifying who is likely to respond to treatment, who shares a similar underlying profile and which dimensions of psychopathology are most biologically salient in a given individual.

Future research would benefit from greater precision in how transdiagnostic biomarkers are defined, operationalized and reported. Studies should specify whether the transdiagnostic approach is across diagnoses, beyond diagnoses or in relation to explicit symptom dimensions or hierarchical models. Methodologically, stronger transdiagnostic claims will require designs with adequate representation across the disorders or dimensions under study, along with direct comparison to diagnosis‐based approaches when possible. Greater attention to replication, external validation and longitudinal prediction will also be important, particularly if transdiagnostic biomarkers are to support prognosis, stratification or treatment selection. Finally, the literature suggests a need to broaden the current emphasis on brain‐based markers by expanding transdiagnostic work on peripheral, neuroendocrine, inflammatory and other multimodal biomarkers that may index shared psychopathology across diagnostic boundaries.

## 5 Conclusion

The literature synthesized in this conceptual review indicates that transdiagnostic biomarker research has emerged as a meaningful response to the limitations of diagnosis‐based psychiatric research. Rather than anchoring biomarker discovery solely to categorical disorders, this body of work links measurable indicators to shared symptom dimensions, cross‐diagnostic phenotypes, burden, vulnerability and treatment‐response patterns. In doing so, it reflects a broader shift towards models of psychopathology that better accommodate heterogeneity, comorbidity and early‐course presentations. This may be especially important for early intervention research, where identifying shared risk profiles and emerging symptom trajectories may help inform earlier recognition, stratification and treatment planning.

## Funding

The authors have nothing to report.

## Conflicts of Interest

The authors declare no conflicts of interest.

## Supporting information


**Table S1:** Characteristics of studies included in the conceptual review of transdiagnostic biomarkers.

## Data Availability

Data sharing not applicable to this article as no datasets were generated or analysed during the current study.
